# Going above and beyond: a qualitative study on the experiences and perspectives of HIV healthcare providers in Greece

**DOI:** 10.1186/s12913-021-07380-6

**Published:** 2021-12-20

**Authors:** Dimitrios Kyrou, Nikolaos Vrontaras, Christina Karamanidou

**Affiliations:** Institute of Applied Biosciences, Centre for Research and Technology Hellas, 6th km Charilaou-Thermi Road, 57001 Thessaloniki, Greece

**Keywords:** HIV, Healthcare, Providers, Perspectives, Qualitative

## Abstract

**Background:**

This study concerns the experiences and perceptions of healthcare providers (HCPs) in Greece, as they respond to the changing health and psychosocial needs of people living with HIV (PLHIV) in unfavorable conditions, within a healthcare system encumbered by a decade of austerity measures.

**Methods:**

To this end, semi-structured interviews were conducted with 20 HCPs in six HIV units throughout Greece. The data were analyzed employing reflexive thematic analysis, under a critical realist approach.

**Results:**

Three main themes were generated from the analysis of the data: 1) Care Beyond Medicine, 2) Compensating System Barriers Towards Optimized Care and 3) Role Appraisal. According to HCPs’ perspectives, 1) their role extends beyond medicine, as they provide care for vulnerable groups and respond to their psychosocial difficulties. 2) Struggling to overcome structural barriers, HCPs often manage to provide privileged care to PLHIV. 3) In doing so, they become excessively involved. Despite the fulfillment experienced, HCPs often feel invalidated by the hospital setting, and frustrated, when they cannot manage to respond to the pressing needs of PLHIV.

**Conclusions:**

HCPs are forced to pull together their personal resources in order to coordinate care and respond to PLHIV’s psychosocial difficulties and health issues, as there is no formal model of coordination of care for PLHIV in Greece. Considering these findings, the development of case management protocols and integrated care pathways in Greece is being proposed.

**Supplementary Information:**

The online version contains supplementary material available at 10.1186/s12913-021-07380-6.

## Background

The advent of antiretroviral therapy (ART) dramatically improved the health outcomes and maximized the life expectancy of people living with HIV (PLHIV), transforming HIV infection into a chronic disease [1]. Along these lines, the needs of PLHIV are changing and increasing in complexity. Chronic HIV care includes the management of co-morbidities, polypharmacy, ageing-related processes, such as frailty, in addition to the heterogeneous components of care for long-term infection [2].

Adequate treatment outcomes depend upon adherence to drug therapy and retention in care [3, 4]. Socioeconomic factors, such as poverty, unstable housing, lack of insurance, mental health issues, substance dependence and experience of HIV-related stigma, have been found to impede engagement in care in developed countries [5–7]. Furthermore, psychosocial stressors (e.g., unemployment, isolation, functional decline) increase with age, further disrupting the retainment of PLHIV in appropriate care [8, 9].

The role of healthcare Providers (HCPs) is of crucial importance for engaging and retaining PLHIV in care. Previous studies on the perspectives of HCPs who work with PLHIV have highlighted the importance of engaging them on a personal level and forming relationships based on trust, empathy, respect and validation [5, 10, 11]. Other important strategies and activities included delivering both clinical services and concrete support, linking PLHIV to other system services, providing education and making active efforts to reach PLHIV (outreach strategies) [11, 12]. Facility-based challenges, such as staffing shortages, large provider caseloads and weak integration of HIV care and other healthcare services, and patient difficulties, such as social disparities and logistic barriers, were designated as barriers to care provision by HCPs in recent qualitative studies [5, 10].

Social disparities are extremely relevant to the HCPs’ role in the provision of HIV care in Greece, as an unprecedented economic crisis and a decade (2009–2018) of austerity measures raised the percentage of the population facing material deprivation to 36% in 2017 [13]. At the same time, public health expenditure severely decreased resulting in reduced hospital funding, overburdened psychosocial services (e.g., the waiting time for opium substitution treatment in Athens was 8 years in 2011) and an increase in the population’s unmet health needs [14–16]. In a recent qualitative study, HCPs in Greece raised concerns regarding their ability to efficiently fulfill their role within a decaying healthcare system, with shortages of equipment and staff. In addition, they underlined the difficulty of access to the healthcare system of vulnerable groups, such as those with chronic diseases, and issues regarding the prioritization of care for certain vulnerable groups [17].

Furthermore, during the last few years the incidence of HIV in Greece increased dramatically. An outbreak of new cases among persons who inject drugs (PWID) occurred in 2011, with a 16-fold increase in this -difficult to treat-population that traditionally accounted for few sporadic cases [18]. According to Vourli et al. [19], PWID were less likely to be virally suppressed, adherent to therapy and linked to care. Overall, according to a recent cross-sectional study in Greece [15], uninsured PLHIV or those who reported lower socioeconomic status were significantly more likely to face obstacles in accessing HIV care, had limited access to laboratory tests, were affected by shortages in ART medicines, were subjected to long waiting times for medical appointments, had difficulties in accessing the hospital pharmacy due to restricted working hours and were less likely to be aware of their viral load.

Against this background, the provision of HIV care by HCPs has been saddled with system-level impediments and increasing psychosocial needs. From this standpoint, critical issues regarding HCPs’ perspectives and their role in HIV care during this challenging phase remain unanswered, i.e., what are their experiences in providing HIV care and retaining PLHIV in care, how do they manage to tackle structural impediments, how do they respond to pressing population needs, how do they appraise their roles, and what would facilitate their work? To the best of our knowledge, this is the first study that captures HIV providers’ perspectives in Greece.

## Method

### Design

This study utilized a qualitative methodology, i.e., reflexive thematic analysis [20–22], to construct and describe coherent patterns (themes) grounded in the data. A critical realist framework was utilized [23], to allow the exploration of the experiences of HCPs as real and true to them, while recognizing the mediating power of social context and individuals’ discrete locatedness and perspectives.

On these grounds, qualitative data were gathered via semi-structured interviews with the healthcare staff of HIV specialized units, to enable the in-depth exploration of HCPs’ experiences and perspectives. Reporting of the study’s methodology and findings adheres to the consolidated criteria for reporting qualitative research (COREQ) [24].

### Setting

In Greece, HIV care is provided in infectious disease clinics. There are 12 clinics in the region around the capital city and a further five in other Greek regions. There are also 9 outpatient clinics that provide care for PLHIV [25]. ART is recommended for all PLHIV, regardless of the CD4 count [26]. Treatment costs are covered by health insurance, or by social welfare and hospital budgets for the uninsured [25].

### Sample

Utilizing purposive sampling, 20 HCPs working in specialized HIV units in six state hospitals in major Greek cities (Athens, Thessaloniki, Alexandropoulis, Patra) were recruited by phone between September 2019 and November 2019. An effort was made to select hospitals situated in regions covering most of mainland Greece from north to south. The sample consisted of 12 infectious disease (ID) specialists, 1 physician, 4 nurses, 2 psychologists and 1 member of administrative staff. Eight [8] were male and twelve [12] were female. They had a mean of 12.1 years of experience working with PLHIV, with a range of 6 months to 32 years (see Table 1).

**Table 1:** Study Participants’ Characteristics

Participant Number	Role	Years of Service in HIV units
11	Physician	6 months
12	Nursing Staff	15 years
13	ID specialist	6,5 years
21	ID specialist	32 years
22	Nursing Staff	6 years
23	ID specialist	13 years
24	ID specialist	21 years
31	ID specialist	15 years
32	ID specialist	11 years
33	ID specialist	11 years
34	Psychologist	11 years
41	Psychologist	21 years
42	Nursing Staff	4 years
43	ID specialist	10 years
44	ID specialist	20 years
51	ID specialist	1,5 years
52	Nursing Staff	4 years
53	Administrative Staff	20 years
54	ID specialist	12 years
61	ID specialist	8 years

Participants’ role and years of service in HIV units

### Material

A semi-structured interview guide was developed for data collection (see Supplement Α). It included discussion points regarding: a) the HCPs’ role in HIV care provision, b) their relationship with the PLHIV they treat and c) the healthcare setting they work in, d) the care pathways. Open-ended questions were formed to facilitate a detailed exploration of participants’ experiences and perspectives.

### Procedure

None of the HCPs refused to participate. The semi-structured, face-to-face interviews were conducted in Greek following the interview guide. Probing questions were asked when necessary in order to obtain a more thorough understanding of the issues raised. All interviews were conducted by one of the researchers (CK), a female health psychologist (PhD) employed as a post-doctoral researcher, with prior training and extensive experience in semi-structured interviewing for research purposes in health psychology. The interviewer had no biases regarding the research questions and no other relationship with the participants, who were only informed that she was a health psychologist.

The interviews took place in an assigned HCP’s office within each HIV outpatient clinic, so that participants’ privacy was secured. They were audio-recorded and had a mean duration of 35 min, with a range of 19 to 55 min. No field notes were taken during or after the interviews, as they were being audio-recorded. Interview transcripts were not returned to participants, and no repeat interviews were carried out, in the interest of analyzing participants’ spontaneous and unstudied articulation of their experiences and perspectives.

Data collection was considered complete when data from participants’ accounts had been analyzed and the conceptual semantic structure derived from the analysis of the data was considered adequate in richness and depth for addressing the research questions and the analytical goals of this study [27].

### Analysis

The data were analyzed thematically [20, 21] within a critical realist framework [23]. Semantic coding was carried out by a member of a research team (DK), a male clinical psychologist (MSc) employed as a research associate, with no prior relationship with the participants and without presuppositions regarding the research questions. The analysis was performed in Microsoft Office Word.

**Fig. 1:**
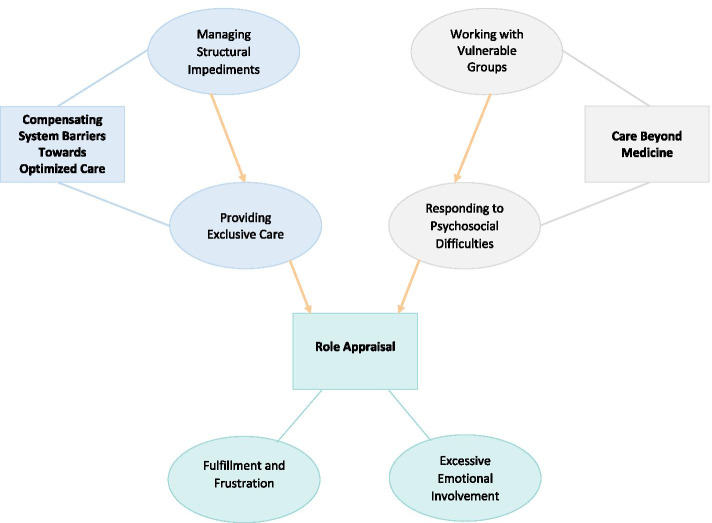
Themes, Subthemes and their Conceptual Relationships. Note: Themes and subthemes are presented in orthogonal and circular shapes respectively. The lines indicate the thematic structure of the data while the arrows indicate conceptual relationships

First, all interviews were transcribed -anonymizing any personal information- and thoroughly read, to achieve familiarization with the data. Then, inductive coding was performed to generate preliminary concepts from the data. Examples of preliminary codes generated from the data were: “feeling close to treated PLHIV”; “taking a psychotherapist’s role to help PLHIV”. Next, the developed codes were collapsed into overarching groups, i.e., subthemes, that reflected repeated conceptual patterns across the dataset. Subthemes were then clustered in higher level groups to form themes that cohered around central organizing concepts grounded in the data. The internal homogeneity and external heterogeneity of the data within subthemes and themes were reviewed in a multidisciplinary research team. Subsequently, subthemes and themes were further defined to better account for the data and to form a cohesive conceptual structure (see Fig. 1). A second member of the research team (CK) was consulting with the first author and thoroughly monitoring the analysis process from initial coding to the development of the final themes.

In order to facilitate the presentation of subthemes and themes, the most indicative transcribed interview quotes were selected. The selected quotes were translated from Greek to English by the main coder (DK) and were checked for comprehensibility by a second member of the research team (CK). Discrepancies in the coding procedure, and in the translation of quotes, were resolved by discussion until consensus was reached.

### Ethical issues

Before conducting the interviews, participants were given information about the purpose of the study and were asked to sign a consent form explicating the protection of their confidentiality, the procedures for data anonymization, storage and treatment, as well as their right to deny answering any of the questions or drop out from the study at any moment. This study was granted ethical approval by the Research Ethics Committee of the Institute of Applied Biosciences at Centre for Research & Technology Hellas (CERTH) and the Research Ethics Committee of each participating hospital. The study was carried out in accordance with the Declaration of Helsinki and is compliant with the European General Data Protection Regulation (GDPR).

## Results

Three themes (i.e., “Care Beyond Medicine”, “Compensating System Barriers Towards Optimized Care”, “Role Appraisal”) and six subthemes were conceptually generated from the data (see Fig. 1). “Care Beyond Medicine” portrays providers’ efforts to respond to a wide range of psychosocial difficulties PLHIV face, as they commonly belong to socially vulnerable groups. These patient difficulties pose impediments to proper healthcare. Besides these patient-level challenges, HCPs also face system-level and facility-level impediments, −e.g., lack of services for psychological support, staffing shortages, discriminations against providing healthcare for PLHIV - which they struggle to manage, as delineated in “Compensating System Barriers Towards Optimized Care”. Accounting for these system-level challenges, often results in “providing exclusive care” to this patient group, which in many aspects is perceived by participants as privileged, compared to the care provided to other patient groups in the current healthcare setting. On the grounds of these structural impediments, patient-level challenges and their struggles to respond to PLHIV’s medical and psychosocial difficulties, participants become excessively involved in caring for PLHIV. Besides their excessive involvement, their “Role Appraisal” entails feelings of fulfillment and frustration, both of which emerge from working as HIV care providers in the current setting.

Themes and subthemes are presented in greater detail below in conjunction with multiple interview quotes, which aim to support and further illustrate the presented concepts. They are also summarized in Fig. 1, along with their conceptual relationships, which are represented by arrows.

### Care beyond medicine

This theme portrays participants’ perceptions regarding the challenges that PLHIV face as well as their experiences in accounting for these difficulties.

According to participants, the treatment of HIV infection has become a manageable task with the advent of new antiretroviral therapies. Nevertheless, HCPs are called to manage PLHIV’s social and psychological challenges.

*“(…) The (current) management of the condition is more complicated. That is to say, it’s not easier due to better medicine or due to the standardization of the process. It’s becoming more and more complicated, and this is due to socioeconomic factors and not due to (the lack of) mere medical knowledge.” (P24, ID specialist, 21 years of service)*

### Working with vulnerable groups

The present subtheme portrays the perceptions of HCPs regarding the challenges that PLHIV in Greece face.

Participants stated that the majority of PLHIV attending the units are men who have sex with men (MSM), PWID and, to a lesser extent, heterosexuals, refugees and prisoners. PLHIV seem to be *“(…*) *an ipso facto special group. They have specific needs and particularities.” (P42, Nursing Staff, 4 years of service).* These particularities are attributed to the HIV stigma and to certain vulnerable social groups, to which most PLHIV belong.

*“I would say that these are more common [in PLHIV] compared to other groups, let’s say, people with cancer, with diabetes mellitus or kidney failure (…) Why is that? Because this is a socially vulnerable group. Thus, this socially vulnerable patient-person will not remain in care” (P24, ID specialist, 21 years of service)*

Participants made reference to the psychological, sexual and work-related difficulties PLHIV experience. Many people with HIV are rejected from their families or refuse to disclose their HIV status, which often destabilizes close bonds with loved ones. As a result, PLHIV carry *“a heavy burden, emotionally.” (P11, Physician, 6 months of service).* In many instances, they even lack the support needed for their medical treatment.

*“These people are most often alone (…) If they don’t have partners, what will happen to them? Their families aren’t aware. Who is, also, going to take care of them? Let’s not fool ourselves. Those difficulties are real.” (P44, ID specialist, 20 years of service)*

Additionally, the serious psychosocial difficulties that many PLHIV face impede proper medical care and their commitment to it.

*“When there are accumulating social issues, you can’t do exactly what should be done, you do less. For example, when there is a homeless person, or an individual without documentation etc., you are sure that this individual will leave the unit and will not take any medication. This person will be roaming around, spreading viral load (…) Models are nice in theory, but there is nothing you can do.” (P24, ID specialist, 21 years of service)*

### Responding to psychosocial difficulties

The present subtheme describes the experiences of HCPs on responding to the psychosocial challenges PLHIV face.

Trying to respond to the particular needs of PLHIV, HCPs’ role becomes *“multidimensional, interactive*.” *(P42, Nursing Staff, 4 years of service).* According to participants*,* a communication style based on trust, validation, acceptance and openness seems crucial for the therapeutic relationship and the health outcomes.

*“I feel that these patients crave to feel accepted. That is to say, because we don’t treat them any different (...) I see they feel very nice, they feel they are in an environment in which they can be free, they know they are not judged. All these things are not found out there.” (P44, ID specialist, 20 years of service)*

Treating PLHIV involves to a large extent the provision of psychological support for issues regarding seropositivity, stigma and personal difficulties.

*“I’m telling you, they need much affection. (...) Between you and me, they mostly need psychological support rather than medical care.” (P43, ID specialist, 10 years of service)*

Taking on this role, two participants stated that they have encountered issues that they could not handle and referred PLHIV to mental health specialists.

*“ (…) There was one case that confided [a traumatic experience] in me. At that moment, I freaked out. I am not a psychologist; I didn’t know how to approach the matter at that moment. I couldn’t handle it myself afterwards, you see, I have found myself in really difficult situations.” (P52, Nursing Staff, 4 years of service)*

Participants mentioned that they also start discussions about sexual safety issues, mostly with MSM, to enable them to protect themselves from other sexually transmitted infections and to keep their partners safe.

*“We try hard to inform them on the transmission routes, on what precautions they can take, on how important it is to take these precautions: ‘Yes, you are in therapy, you are virally suppressed, but that doesn’t mean that you can start having an unrestricted sexual life without caring about any precaution. (…) As you may miss out a pill or whatever, you may transmit it. But, besides transmitting it, other sexually transmitted infections also exist. Taking HIV medication doesn’t mean you are fully protected. Hepatitis, syphilis also exist.” (P11, Physician, 6 months of service)*

Another crucial aspect of HCPs’ role, according to participants, is the interconnection with other care structures so that PLHIV can receive psychosocial support and material assistance.

*“Giving them medication and enrolling them in rehabilitation centers is not the problem. You have to support them. Meaning, if you have a homeless, a desolate, a refugee who has no stake in the future and lives in the park, prescribing him medication is not the problem. The problem lies in housing, providing food, helping, referring them [to services]. (P44, ID specialist, 20 years of service)*

Some participants noted that while working in HIV units, they have been subjected to demanding, manipulative, rude and even aggressive behaviors by PLHIV or their relatives, which they try to put limits to.

*“I have been shocked by parents’ [of PLHIV] extreme behaviors, there have been beatings during the sessions (…) they have pushed me (…) I have even been intimidated by my own patients during sessions.” (P34, Psychologist, 11 years of service)*

Regarding the management of poor adherence, participants said they spend time discussing the necessity of medication and the acceptance of seropositivity, particularly with women and people who acquired the virus vertically.

*“One other woman I treat gave birth to a child, (...). She has a very resistant virus. We managed to carry the pregnancy to term with great difficulty. I needed to see her very regularly during pregnancy, mostly to … make sure that she takes her medicines and that she does it correctly. At least to prevent her child from being infected.” (P54, ID specialist, 12 years of service)*

Some participants claimed that they actively search PLHIV who abandon medical care *“by calling home or contacting relatives” (P22, Nursing Staff, 6 years of service).*

Lastly, participants emphasized the contribution of HIV units’ psychologists to every aspect of PLHIV’s difficulties and expressed the need for mental health specialists in units that do not currently employ such personnel.

### Compensating system barriers towards optimized care

Participants’ challenges go beyond the distinct and multifaceted needs of PLHIV. HIV units’ HCPs are confronted with impediments stemming from the broader hospital setting and the healthcare system. In the end, participants manage to provide PLHIV with a quality of care that is often privileged compared to the care provided to the general population and other patient groups.

### Managing structural impediments

The present subtheme portrays the perceptions and experiences of HCPs regarding an array of system-level and facility-level barriers they face.

As illustrated in the quote below, the rising number of PLHIV has not been followed by hospital structures’ corresponding expansion.

*“Time goes by. There are increasingly more people. Individuals, difficult to manage, are added in care. We don’t have the proper background to provide such care, nor the proper facilities, and often not the proper support (…) A confluence has occurred, but our work structures haven’t evolved accordingly. They may even be shrinking because it coincided, I mean, with the crisis. Thus, there lies the difficulty.” (P24, ID specialist, 21 years of service)*

Participants mentioned that their role is challenged by shortfalls in physicians, administrative staff and mental healthcare specialists. As a result, HCPs work under time pressure and, often, beyond their work shifts. In addition, they take over duties that do not pertain to their specialty, which consequently restrains them from providing optimal clinical care:

*“Objectively, during this time I don’t do what I’m supposed to (…) because there wasn’t anyone else to help, I did. So, I inevitably left my part fall short (…) If things were better and there was administrative support and we didn’t need to be occupied with other tasks, yes, I believe that my nursing duties would be fulfilled.” (P22, Nursing Staff, 6 years of service)*

Problems in the interconnection with psychosocial services put an extra burden on participants, pressuring them to provide care beyond their specialties. The interconnection regarding HIV infected prisoners and migrants is subjected to long delays and lack of interpreters, resulting in problematic and time-consuming communication, while the interconnection with substance abuse services is either absent or scarce.

Participants stated that on many occasions they were compelled to deal with emerging issues in alternative ways, due to the lack of corresponding support structures for PLHIV who face social difficulties.

*“At this point I keep two migrants in the hospital for months. Because they have nowhere to go! The system is blocked, and I don’t know why! I may sound funny, that is to say, I have even approached a patient of the same nationality, who has a home, and asked ‘Can you perhaps host them?’ (...) operating completely outside the system.” (P24, ID specialist, 21 years of service)*

Additionally, the monthly manual prescription of medicines, the dysfunctional bureaucratic system and the frequent encounter of administrative issues add to HCPs’ workload and increase their fatigue. Participants expressed the need for the simplification and advancement of processes regarding patient registration, drug prescription and application for the HIV benefit.

*“What mostly tires us out, are administrative procedures. Bureaucracy, in general. Granting that these people have to come and pick up their medicines every month, and there is often a lack of medicines, which is tragic because it puts a heavy load on us compared to … they could be visiting two times per year and take their medicines altogether to get this over with and make everybody’s life easier. In order to order drugs, we need to prepare a tremendous amount of paperwork, which we don’t know how to send. Silly bureaucratic procedures are what mostly tires us out.” (P23, ID specialist, 13 years of service)*

Some participants described shortfalls in drugs and viral load tests, which led them to use obsolete clinical markers in their clinical practice and to engage in social actions e.g., distributing pills, contacting TV stations and putting pressure on hospitals’ administrations to restore the shortcomings.

*“I have experienced times when there were shortages of medicines. And I was trying to distribute the pills so nobody was left without ( …) Anyways, we were opening the bottles and we were somehow allocating the pills. I had looked for bottles [with pills] at the ‘Doctors of the World’ and I had found some (…) We had brought TV reporters here [at the hospital] regarding drug shortages (…)” (P52, Nursing Staff, 4 years of service)*

Another difficulty mentioned by multiple participants was that many physicians outside HIV units refuse to provide medical care for PLHIV.

*“Nobody wants to get involved [in the care of PLHIV]. Everybody turns their back, making up excuses for not doing what should be done (…) Not everyone is like that. There are those without such reservations that want to be involved and they do so. (…) But I think most of them [HCPs outside HIV units] want to wash their hands of them [PLHIV].” (P51, ID specialist, 1,5 years of service)*

### Providing exclusive care

This subtheme refers to participants’ experiences in accounting for numerous structural impediments, paradoxically resulting in exclusive care provision for PLHIV.

More specifically, discriminations in the provision of care for PLHIV by a large portion of physicians, means that ID specialists need to manage medical conditions which: *“sometimes exceed our own specialty (…), exceed the subject of infectious diseases, becoming a complex clinical practice.” (P31, ID specialist, 15 years of service).* One participant outlined:

*“So, when someone bleeds (…) from the gastrointestinal tract and the emergency department’s physician says ‘Go tomorrow morning to your doctor’! And he refers him here, to this clinic, where you cannot treat this, and without providing the treatment that others would have had. That is a problem. And that puts a load on you (…). Since others don’t take them on, you are the one who has to. And how are you going to manage something that’s out of your hands?” (P23, ID specialist, 13 years of service)*

Two participants mentioned that PLHIV are even given the right to attend the units without an appointment in emergency situations, instead of going to the emergency department.

HIV unit physicians face the extra burden of connecting and scheduling PLHIV’s medical appointments with physicians outside the clinic. This first-hand communication with physicians of other specialties adds to participants’ difficulties and workload.

*“Besides, those patients, just because they feel this terrible stigma and don’t want to confide their seropositivity to others, they want everything to take place in our units. That is, to see an endocrinologist to whom they can tell they are seropositive, to see a cardiologist who thus … and many times they don’t do this on their own. It’s been a month and a half now, that we have been trying to liaise between this patient and a surgeon. And now we have found one, he rescheduled many times, we found a new surgeon and we are experiencing the same situation all over again (…). So, the workload is heavy and at the same time I am compelled to put myself through a process which surpasses the scope of my responsibilities, I’m not the one who has to do this.” (P54, ID specialist, 12 years of service)*

One participant emphasized the need for *“a network of physicians with various specialties that could manage these people.” (P23, ID specialist, 13 years of experience).*

In addition to the efforts to liaise between PLHIV and physicians of other specialties, participants often need to find solutions to safeguard PLHIV’s privacy during medical appointments and medication pick-ups. Some participants also mentioned that the HIV units help PLHIV by processing the paperwork for the HIV benefit and mailing medicines to people who live in remote districts.

Ultimately, carrying a disproportionate load, participants manage to provide privileged care to PLHIV, as illustrated in the quotation below:

*“The general population doesn’t have the equivalent provision of medical services as PLHIV do. They have very good provision of medical services, very very good, exceptionally good ( …) We are the ones to always arrange things in order to make it easier for them. This is not the case with the general population (…). This system is to blame, it’s a system’s deficiency which we are left to handle and they are benefitting. Thus, they receive much better services, I wish we could provide all of my other patients the same services.” (P33, ID specialist, 11 years of service)*

### Role appraisal

The present theme portrays participants’ appraisal of their role, including its emotional impact, and their self-reflections regarding their personal and professional stance towards PLHIV that they treat.

#### Fulfilment and frustration

This subtheme portrays providers’ feelings of fulfilment and frustration which emerge from working in HIV units.

Participants expressed that they feel satisfaction working in HIV units as they find the medical management of HIV infection and comorbidities interesting. In addition, they feel satisfied and moved as they work to restore PLHIV’s physical health and social functionality.

*“Those people exist, to whom you can give strength to overcome the news, the bad news they receive, to return to a normal way of life and continue to have a normal life, a productive one. To have children, which was prohibited for them in the past. Of course, I get great satisfaction and joy.” (P21, ID specialist, 32 years of service)*

Apart from medical management, participants gain satisfaction by contributing to PLHIV’s psychosocial support. Some participants mentioned that through their jobs they had new social experiences, matured mentally and started to know themselves better.

*“It [the job] offered me a lot. It changes you as a person, it makes you come in contact with people you’d never believe you would, (…) such as, drug users (…) I mean to tell you that (...) you come face to face with your inner self and who you think you are. It’s a very useful experience, I would say.” (P44, ID specialist, 20 years of service)*

However, working in HIV units also entails feelings of frustration. On some occasions, physicians are unable to provide support for PLHIV’s psychosocial needs. Moreover, despite participants’ efforts, some PLHIV abandon care in the end.

In spite of the friendly and supportive cooperation among HCPs in HIV clinics, participants often feel frustrated with and invalidated by the broader hospital setting, as aptly delineated below:

*“And the physician is the one ultimately pounded by a rogue wave, which, ‘boom’, punishes him. The system punishes him. Because, it says, ‘you brought this filthy, dirty (...)’. The structures punish him because they attribute the patient to him, and if the physician (...) hasn’t the ability … to not take it personally he will enter an atrocious tug-of-war. The structures punish him, and the patient himself punishes him. Because the patient feels the physician is the one to blame, as he is the one who the patient sees! (…) So, he’s punished from both sides, which are substantially the same thing in different forms.” (P24, ID specialist, 21 years of service)*

#### Excessive emotional involvement

The present subtheme describes the participants’ feelings of closeness to PLHIV they treat, as well as their inner negotiations regarding setting boundaries.

The chronicity and nature of medical attention for PLHIV cultivates *“a contact, which is closer” (P44, ID specialist, 20 years of service*), resulting in the formation of emotional bonds between physicians and PLHIV.

*“I have known many PLHIV for 10 years now and... I’m aware of many of their personal difficulties. For instance, they have lost their parents, they have divorced. And they share all these things every time, so a more personal relationship is established and not strictly a professional one- for better or worse.” (P23, ID specialist, 13 years of service)*

Participants reported warm and pleasant feelings, such as happiness and completeness, ensuing from the emotional closeness with PLHIV. On the other hand, participants talked about their intense sadness in cases of medical complications and the passing of people they treat.

*“I’ve also grown attached to them. It isn’t good for you to become too attached (…) We have been to the funerals of those who have died, we have cried like we were crying for our close ones. But it feels they were our close ones. And when some are seriously ill, we are sad and think about it at home. It’s impossible not to get too attached in this line of work. (...) Could anyone leave this place and press the delete button (…)? I cannot.” (P12, Nursing Staff, 15 years of service)*

Furthermore, some participants mentioned moments of mental exhaustion due to excessive involvement in PLHIV’s care.

*“With many patients I go above and beyond. And this is the reason why I often become mentally exhausted. It’s like I am trying to help beyond the boundaries within I am allowed to move.” (P54, ID specialist, 12 years of service)*

Finally, a number of participants referred to their efforts in putting boundaries to themselves regarding PLHIV’s care and the emotional impact of their roles.

*“Regarding my own personal growth, my goal is to establish boundaries, seeing that many times I overstep them. At a personal cost.” (P54, ID specialist, 12 years of service)*

## Discussion

In summary, as HCPs make efforts to respond to PLHIV’s growing psychosocial difficulties, their role becomes multifaceted and extends beyond medical care. Providing HIV care in Greece has become challenging due to the lack of resources, administrative and operational problems as well as barriers to linkage to psychosocial services. Rising to the occasion can be fulfilling for HCPs, but it does not come without a cost.

Utilizing frontline HCPs’ perspectives, this study validates empirical data of previous research regarding HIV care provision in Greece [15, 19, 28]. In addition, broader system-level issues mentioned in the present study, such as staffing shortages, time pressure, inefficient policies, and patient barriers, e.g., housing instability, have been also identified by HCPs working in different countries [5, 10]. HCPs’ perspectives on tackling these challenges in Greece show similarities with perspectives documented in other countries. These include the emphasis of a client-centered patient-provider relationship based on trust and accessibility, the provision of psychosocial support, outreach activities, assistance with system navigation and interconnection with needed services [5, 11]. The similarities between HCPs’ challenges and responses in HIV care worldwide are striking, and ultimately reduced to the management of at-risk populations within unaccommodating health systems [2, 5, 10, 11, 29].

What emerged as a particular finding in the present setting was that the efforts to safeguard PLHIV’s care against an array of system-level and patient challenges, resulted in a form of care that was perceived by HCPs as privileged, compared to the care provided to other patient groups. Moreover, HCPs’ excessive emotional involvement in caring for PLHIV was a source of both fulfillment and frustration for them, according to their perspectives.

Numerous strategies have been proposed and implemented to facilitate HCPs’ role and advance care for PLHIV, such as the creation of medical homes and the adaptation of the Expanded Chronic Care Model for HIV care [2, 3, 5, 30, 31]. Regarding resource poor settings, relocating ART distribution, symptom monitoring and psychosocial or health-related support from health facilities to communities (Community-based service delivery) has been shown to improve retention in care [32]. Additional models implemented in resource poor settings include the decentralization of HIV service delivery from tertiary care centers to lower-tier health facilities and delegating routine HIV care from over-burdened physicians to other HCPs (task-shifting) [33]. Differentiated Service Delivery models, i.e., models adjusted to specific requirements, preferences and needs of subgroups of clients along the cascade (e.g., more frequent contacts with HCPs for those with low CD4 on ART initiations), are also utilized in low-income countries, for better allocating health system resources and improving long-term retention [32, 34].

However, according to our findings, there is no formal model of coordination of care for the complex needs of PLHIV in Greece. As a result, HCPs are forced to pull together their personal resources (social, psychological, etc.) in order to coordinate care and respond to PLHIV’s psychosocial difficulties, as well as additional health issues beyond the management of the infection. This kind of informal management does not always lead to the desired outcomes and accounts for a great source of stress and frustration for HCPs, while also adding to their workload.

### Recommendations

Along these lines, the development of integrated care pathways [35] could facilitate efficient system navigation and reduce HCPs’ burden by formally structuring coordinated care for PLHIV in Greece. In addition, physicians’ burden could be significantly reduced by defining care protocols for shifting some of the less complex components of care to other members of the healthcare team [32, 33]. Moreover, defining case management and HCP training protocols, with a focus on psychosocial assessment, making appropriate referrals and promoting the utilization of informal supportive networks, could yield better outcomes for PLHIV and facilitate HCPs’ role. Also, encouraging PLHIV to create peer-support navigation networks could assist them in utilizing available clinical and psychosocial resources, increase PLHIV’s activation and empowerment and reduce HCPs’ workload. Moreover, as HIV care delivery is associated with a high risk of burnout [36, 37], interventions aimed at improving work-life balance could be notably supportive and moderate HCPs’ excessive emotional involvement.

As suggested from participants’ accounts, the simplification of processes regarding the HIV benefit and the medical prescriptions (i.e., annual prescription of medicines instead of monthly) could reallocate the time spent on bureaucracy to care provision. Lastly, interventions to raise awareness on caring for PLHIV among HCPs of various specialties and roles in the healthcare system is crucial to the present setting.

### Limitations and future research

Despite the inclusion of hospitals from both large cities and smaller towns, the number of participating HIV units potentially limits the generalizability of this study’s findings.

Building on these findings, future research may investigate PLHIV’s satisfaction with care as well as the perspectives of high-level members of management and HCPs in various specialties in hospitals with HIV units. Gaining a broader picture from all involved actors might act as a powerful driver for improving HIV care delivery in Greece.

## Conclusion

Aiming to respond to PLHIV’s health and psychosocial needs and manage structural impediments, HCPs are struggling with challenges, which often surpass the scope of their professional responsibilities. In order to provide quality care to PLHIV in the specified healthcare context, HCPs are utilizing informal strategies and accessing personal resources. The development of formal strategies to coordinate care for PLHIV in Greece, would have the potential to significantly support and facilitate HCPs’ role, as well as improve health and psychosocial outcomes of PLHIV.

## Supplementary Information

**Additional file 1..**

## Data Availability

The datasets generated and analyzed during the current study are not publicly available due to the fact that the interview transcripts are in participants’ native language, i.e., Greek, and therefore not suitable for deposit in a public repository. A de-identified dataset may be made available upon reasonable request from the corresponding author.

## Abbreviations

ART: Antiretroviral Therapy
CERTH: Centre for Research & Technology Hellas
COREQ: Consolidated Criteria for Reporting Qualitative Research
GDPR: General Data Protection Regulation
HCP: Healthcare Provider
HIV: Human Immunodeficiency Virus
ID Specialist: Infectious Disease Specialist
MSM: Men who have Sex with Men
PLHIV: People Living with Human Immunodeficiency Virus
PWID: People Who Inject Drugs
