# Supplementary material for: Going above and beyond: a qualitative study on the experiences and perspectives of HIV healthcare providers in Greece
Source: BMC Health Serv Res. 2021 Dec 20;21:1358. doi: 10.1186/s12913-021-07380-6 (PMC8686363; doi:10.1186/s12913-021-07380-6)
Supplement: Supplementary file 1 — Additional file 1.. [file 12913_2021_7380_MOESM1_ESM.docx]

SUPPLEMENT A: INTERVIEW GUIDE

**Interview Guide**

**Role**

1. How would you appraise your overall experience as a/an ___________ (ID specialist, nurse, psychologist, administrative staff) in HIV care?
2. Are you satisfied with this role?

**Relationship with PLHIV**

1. What is your experience regarding interacting with PLHIV?
2. Have you encountered challenges while communicating with PLHIV?

**Healthcare System**

1. Could you describe your working experience as a __________ (ID specialist, nurse, psychologist, administrative staff) in this specific unit?
2. Could you describe some of the difficulties you have encountered in this specific healthcare environment?

**Care pathway**

1. Could you describe in detail the clinical pathway for individuals who test positive for HIV?

Prompts

- Opinion about diagnosis announcement.

1. After diagnosis, when is treatment initiation discussed?
2. What are the reactions of PLHIV during these discussions?
3. Would you personalize this procedure according to patients’ characteristics (e.g., MSM, PWID, etc.)?
4. What is the healthcare plan after the initiation of treatment?

Prompts

- Tests, examinations

1. How often do PLHIV come to the unit?

Prompts

- Own initiative
- Scheduled appointments

1. What are the current conditions of care provision for older PLHIV or people who have lived with HIV for longer periods?

Prompts

- Health issues
- Test, examinations
- Appointments
- Long-term treatment
